# Posterolateral Instrumented Fusion in Elderly Patients With a Single Osteoporotic Vertebral Fracture: Evaluation of Health-Related Quality of Life

**DOI:** 10.7759/cureus.53498

**Published:** 2024-02-03

**Authors:** Stylianos Kapetanakis, Nikolaos Gkantsinikoudis, Sotirios Apostolakis, Paschalis Tsioulas, Constantinos Chaniotakis

**Affiliations:** 1 Department of Minimally Invasive and Endoscopic Spine Surgery, Athens Medical Center, Athens, GRC; 2 Spine Department and Deformities, Interbalkan European Medical Center, Thessaloniki, GRC; 3 Orthopaedics, Papageorgiou General Hospital, Thessaloniki, GRC

**Keywords:** health-related quality of life, thoracolumbar junction, neurological deficit, posterolateral instrumented fusion, osteoporotic vertebral fractures

## Abstract

Introduction

Treatment of osteoporotic vertebral fractures (OVFs) is a factor that affects the quality of life and should be considered during management. In patients with a single OVF and neurologic deficit, surgical procedures aiming at neural decompression with instrumented fusion should be considered in elderly individuals. Posterolateral instrumented fusion (PLF) constitutes a largely performed fusion surgery for patients featuring indications for fusion surgery. The aim of this study was to determine the safety, effectiveness, and impact on health-related quality of life (HRQoL) of PLF surgery in elderly patients diagnosed with a single OVF.

Methods

This study was conducted at Interbalkan European Medical Center, Thessaloniki, Greece. Eighty (80) consecutive individuals with OVFs were subjected to PLF and recruited in this prospectively designed non-randomized study. Clinical evaluation was performed preoperatively and postoperatively at particular chronic intervals at one, three, six, and 12 months and two years. The assessment was conducted via the standardized Visual Analogue Scale (VAS) and Short-Form 36 (SF-36) Medical Health Survey Questionnaire for pain and HRQoL, respectively.

Results

No major perioperative complications were observed. All parameters of SF-36 presented significant improvement over the entire follow-up period with VAS scores reaching a plateau at six months. Depicted improvement of these parameters proves the beneficial role of PLF in elderly patients who suffered from a single OVF with or without referable neurological deficit.

Conclusion

OVFs have a significant impact on the quality of life of elderly patients, and surgical treatment with PLF with or without decompression can lead to functional recovery, pain relief, and HRQoL amelioration. Our results demonstrated that the outcomes of PLF in the surgical treatment of these patients are remarkably favorable, demonstrating the safety and efficacy of the technique.

## Introduction

Osteoporosis is the most common metabolic bone disease worldwide [1]. Osteoporotic vertebral fractures (OVFs) constitute the most frequent type of osteoporotic fracture, featuring a prevalence of 15% in women over 50 years old [1]. The number of approximately annually occurring OVFs is calculated at 550,000 worldwide [2]. An OVF, as a strong predictor of morbidity, represents a frequent reason for admission in elderly patients, significantly affecting patients’ quality of life due to resulting back pain and associated deformity. The most frequent OVF site is the thoracolumbar junction (T12-L2), followed by the mid-thoracic spine [3].

The management of OVFs remains a veritably controversial issue in the global spine community [4]. The primary treatment option includes conservative treatment (analgesic regimen, braces, and anti-osteoporotic drugs with dietary recommendations), in an attempt to relieve pain, restore affected segment biomechanics, and reverse the physical course of underlying diseases [3,4]. Surgical intervention has specific indications, including severe persistent pain (painful nonunion), spinal deformity (progressive kyphosis), and the emergence of neurological deficits [3,4]. Progressive collapse of an OVF's vertebral body is demonstrated in 30% of patients. Nevertheless, the incidence of underlying neurologic compromise is reported to be relatively low, encountered in 3% of cases [5].

Vertebroplasty and kyphoplasty, as percutaneous minimal invasive surgical techniques with cement augmentation into the vertebral body, represent suitable interventional treatment methods for patients with persistent pain at the fracture site [6]. Patients with neurologic deficits are not candidates for these techniques, and surgical procedures aiming at neural decompression with instrumented fusion should be considered in these individuals [7]. Posterolateral instrumented fusion (PLF) constitutes a largely performed fusion surgery for patients with OVFs featuring indications for fusion surgery [5,8].

Investigating the safety and efficiency of PLF for the surgical management of OVFs, postoperative pain status, and biomechanical profile of operated spine segments has been studied in specific published literature reports [9-11]. However, to our best knowledge, there is a scarcity of data in recent literature investigating the holistic impact of PLF in health-related quality of life (HRQoL) in patients with OVFs.

The aim of this study is to determine the safety and effectiveness of PLF surgery in elderly patients diagnosed with a single OVF. The prospective design adopted a follow-up interval analysis, and the evaluation of HRQoL underlines the originality of our study.

## Materials and methods

Population and approvals

**Figure 1:**
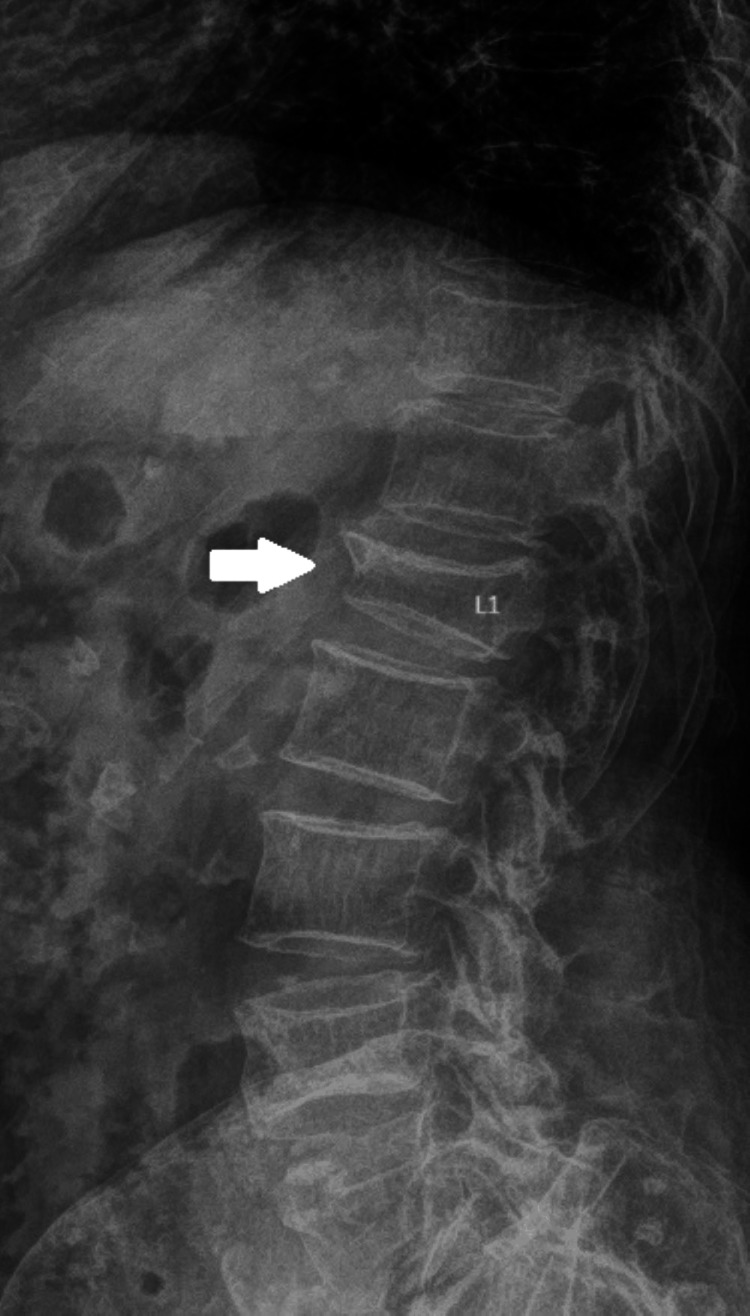
Preoperative radiograph (lateral view) of the lumbar spine. The white arrow depicts the osteoporotic vertebral fracture (OVF) of L1.

All consecutive patients enrolled in this study were diagnosed with OVFs with clinical and radiological evaluation, fulfilling current indications from fusion surgery (Figure 1). All surgical operations were conducted by the same experienced spine surgeon in the same tertiary center. All patients were thoroughly informed about the precise study rationale and design, providing their written consent for participation. Protection of distinct patients’ rights and privacy was strictly warranted during the study performance. Furthermore, the protocol of this study was examined and approved by the Institutional Review Board of the involved hospital (Interbalkan European Medical Center, Thessaloniki, Greece; approval number: 28.12.2016). All aspects of this study were in absolute concordance with the Ethical Principles for Medical Research Involving Human Subjects, as defined in the Declaration of Helsinki of 1964 and its later amendments (2013).

Inclusion and exclusion criteria

The enrollment of patients in this study was decided to specific predetermined inclusion and exclusion criteria (Table 1).

**Table 1:** Inclusion and exclusion criteria of the present study OVF: osteoporotic vertebral fracture

Inclusion criteria	Exclusion criteria
Single vertebral fracture in patients with known osteoporosis	Spine tumor or infection
OVF level from T4 to L5	High-energy spine trauma
Elderly patients [12]	Congenital spine deformity
Presence of clinical or radiologic signs of segmental instability	Multiple spine fractures
Presence of neurologic deficit referrable to fracture	Previous surgery in the affected level

Methods

Eighty (80) patients (total sample) enrolled in this study were subjected to PLF due to having a single OVF from 2019 to 2020. Preoperative neurologic assessment revealed neurologic deficits in 50% of the patients, with the presence of cauda equina syndrome being identified in 5% of the patients. Forty-four (44) patients underwent early surgical treatment, being treated in the first month after fracture clinical emergence, while the remaining thirty-six (36) were subjected to delayed management, after the first month. Clinical evaluation was performed preoperatively and at distinct follow-up intervals at one, three, six, and 12 months and two years postoperatively. The assessment included clinical examination and implementation of well-established patient-reported outcome measures, such as the Visual Analogue Scale (VAS) and Short-Form 36 (SF-36) Medical Health Survey Questionnaire, for the HRQoL analysis. Evaluation of the enrolled patients was performed in the outpatient clinics of the tertiary center within each follow-up checkpoint.

Surgical technique

**Figure 2:**
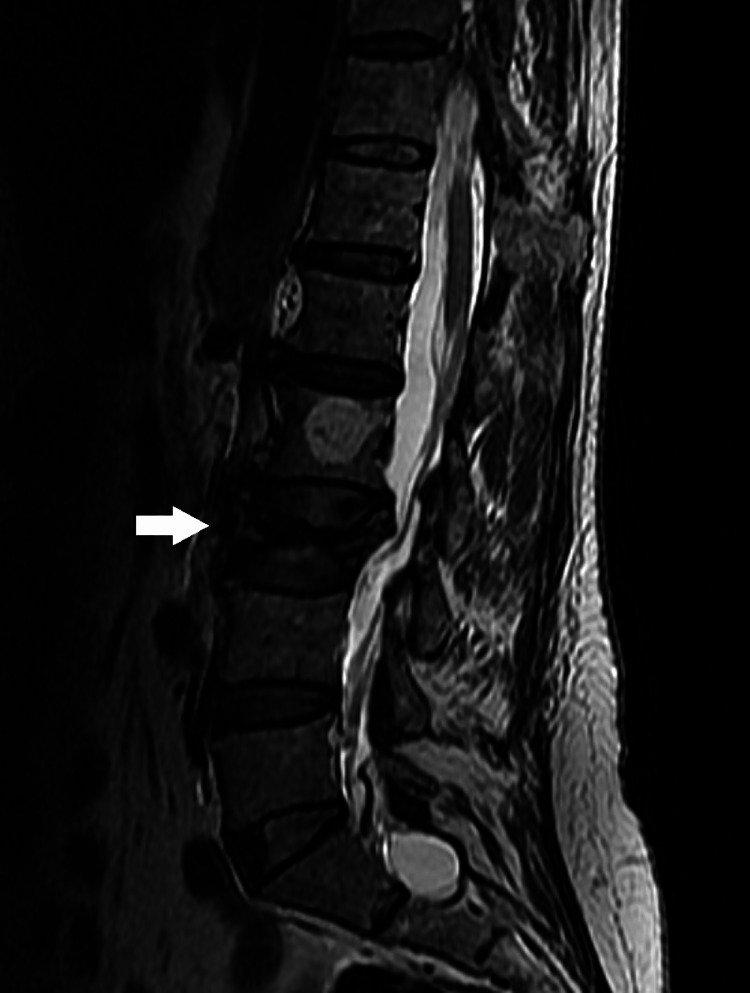
Preoperative lumbar MRI (saggital T2 view). The white arrow depicts the osteoporotic vertebral fracture (OVF) of L3.

**Figure 3:**
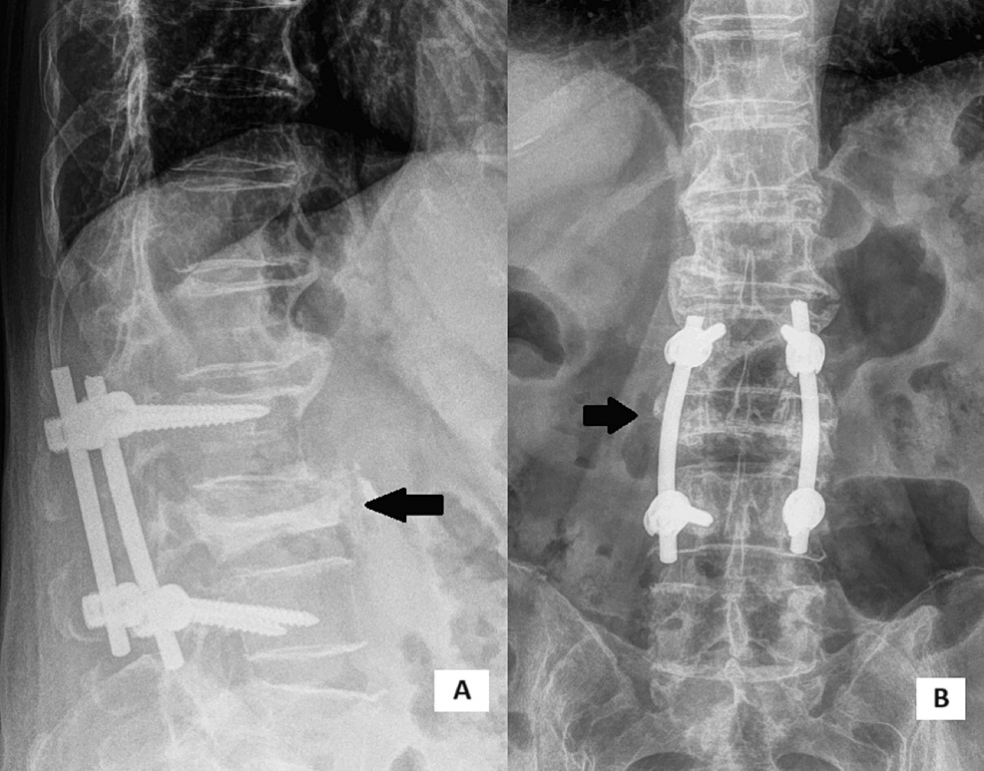
Postoperative radiographs of posterolateral instrumented fusion (PLF) (L2 to L4). The black arrow presents the OVF of L3. Lateral (A) and anteroposterior (B) views.

All patients were subjected to preoperative assessment with magnetic resonance imaging (MRI) and plain radiographs for surgical planning (Figure 2). A standard open posterolateral short-segment fusion with transpedicular screw fixation one level below and one level above the fracture site was conducted in all individuals, as previously described [13]. The whole procedure was performed under constant fluoroscopic navigation with C-ARM in anteroposterior (AP) and lateral views. Briefly, patients were placed in a prone neutral position on a Jackson table under general anesthesia. After level of operation identification with fluoroscopy, a standard posterior approach with skin incision, dissection of subcutaneous tissue, and subperiostal dissection of paravertebral musculature bilaterally was performed. After the dissection of facet joints and identification of transverse processes, posterior transpedicular fixation of the superior and inferior to fractured vertebrae was performed under constant fluoroscopic navigation. Whenever indicated according to clinical and radiologic criteria, posterior decompression with laminectomy-flavectomy and foraminotomy was performed. Fusion was completed with the introduction of titanium rods (6 mm) and autologous bone alongside hydroxyapatite putty, and wound closure was routinely performed by layers (Figure 3). All patients were immediately postoperatively neurologically evaluated, being subsequently transferred into the monitoring chamber for 30 minutes prior to ward return. Finally, they were mobilized with lumbar orthosis on the first postoperative day, being thereafter discharged.

VAS

The VAS is a simple, illustrative method for evaluating various parameters, including pain. In this study, two distinct measurements for lower limb (VAS-leg pain (VAS-LP) and low back pain (VAS-back pain (VAS-BP)) pain were harvested. A horizontal line of 100 mm was utilized in the present study. The patients were asked to indicate their subjective perception of pain with a mark. The level of minimal clinically significant change was designated to be 9 mm. Other parameters (e.g., sex, age, and etiology of pain) were not considered separately [14,15].

SF-36 Medical Health Survey Questionnaire

The SF-36 Medical Health Survey Questionnaire represents a widely used method for evaluating the HRQoL after spine surgery [16,17]. This questionnaire consists of 36 items evaluating eight parameters reflecting patients’ general health: physical function (PF), role physical (RP), body pain (BP), general health (GH), energy, fatigue, and vitality (V), social function (SF), role emotional (RE), and mental health (MH). Each patient was asked to complete the appropriate questionnaire at each regularly scheduled follow-up interval. Responses were collected and converted into percentage scales. A higher score is generally associated with an enhanced HRQoL. A questionnaire was considered invalid if less than half of the entries were completed [15].

Statistical analysis

Statistical analysis was conducted using STATISTICA 10.0 (StatSoft, Inc., 1984-2010) and MATLAB 2016 (The MathWorks, Inc., 2016). Figures were created using MATLAB 2016 and Adobe Illustrator CS3 (Adobe Systems, 2007).

For non-parametric variables, Chi-square, Mann-Whitney’s test, and Kruskal-Wallis H test were used to test for differences between two and multiple groups, respectively. When paired data were compared, the Wilcoxon matched-pair test and Friedman's analysis of variance (ANOVA) were conducted. Spearman’s test was applied to examine for potential correlations between various parameters. Multiple regression was used to investigate for the potential effects of multiple parameters on the outcome measures. In all cases, the level of statistical significance was p < 0.05. The Bonferroni correction for multiple comparisons was used accordingly.

## Results

In total, 80 patients were included in the study. The demographic features of the enrolled individuals are presented in Table 2. A statistically significant difference in gender distribution from that of a theoretical random sample was observed (X^2^_1,80 _= 13.09, p < 0.01). No statistically significant difference in the age profiles of the genders was found (U_1,80 _= 490.5, p = 0.44).

**Table 2:** Demographics of the patients included in the present study.

Demographic features of the patients	
Number of patients	80
Age mean (standard deviation (SD))	78.52 (6.11)
Age median (Min-Max)	78.5 (66-88)
Male (%)	18 (22.5%)
Mean age - male (SD)	79.28 (7.127)
Mean age - female (SD)	78.3 (5.826)

All the patients were subjected to uneventful PLF, featuring no major perioperative complications. Wound infection was present in two patients (2.5%). Oral antibiotics (clindamycin and ciprofloxacin) were used to treat these infections (a total duration of 21 days) without further complications. The mobilization was initiated on the day of the surgery, and all patients were discharged 24 hours after the surgery. Furthermore, all patients successfully completed the intended two-year follow-up.

As far as the individual indices of SF-36 are concerned, a statistically significant difference in the self-reported questionnaire was observed in all parameters. This statistically significant amelioration was also observed for VAS-LP and VAS-BP indices (Table 3).

**Table 3:** Results from Friedman analysis of variance (ANOVA) for the individual parameters of outcome measures. physical function (PF), role physical (RP), bodily pain (BP), general health (GH), energy, fatigue, and vitality (V), social function (SF), role emotional (RE), and mental health (MH), Visual Analogue Scale (VAS), VAS-leg pain (VAS-LP), VAS-back pain (VAS-BP)

Results of Friedman analysis of variance (ANOVA)	
PF	F = 0.99, P < 0.001
RP	F = 0.99, P < 0.001
BP	F = 0.97, P < 0.001
GH	F = 0.97, P < 0.001
V	F = 0.99, P < 0.001
SF	F = 0.99, P < 0.001
RE	F = 0.97, P < 0.001
MH	F = 1, P < 0.001
VAS-LP	F = 0.46, P < 0.001
VAS-BP	F = 0.94, P < 0.001

Post-hoc analysis of the various follow-up checkpoints revealed a continuous improvement of all indices of SF-36 over the entire follow-up period (Table 4). On the other hand, VAS was demonstrated to reach a plateau at six months, featuring no further clinical improvement subsequently.

**Table 4:** Results from the Wilcoxon matched pairs test comparing each index at two successive time points of assessment. Level of significance following Bonferonni correction was determined at p < 0.01. preo: preoperative, mo: month, yrs: years

Post-hoc analysis of the various follow-up checkpoints	
PFpreop vs. PF1mo	Z = 7.77, p < 0.001
PF1mo vs. PF3mo	Z = 7.77, p < 0.001
PF3mo vs. PF6mo	Z = 7.77, p < 0.001
PF6mo vs. PF12mo	Z = 7.77, p < 0.001
PF12mo vs. PF2yrs	Z = 5.16, p < 0.001
RPpreop vs. RP1mo	Z = 7.77, p < 0.001
RP1mo vs. RP3mo	Z = 7.77, p < 0.001
RP3mo vs. RP6mo	Z = 7.77, p < 0.001
RP6mo vs. RP12mo	Z = 7.77, p < 0.001
RP12mo vs. RP2yrs	Z = 5.44, p < 0.001
BPpreop vs. BP1mo	Z = 7.77, p < 0.001
BP1mo vs. BP3mo	Z = 7.77, p < 0.001
BP3mo vs. BP6mo	Z = 7.77, p < 0.001
BP6mo vs. BP12mo	Z = 6.74, p < 0.001
BP12mo vs. BP2yrs	Z = 5.23, p < 0.001
GHpreop vs. GH1mo	Z = 7.77, p < 0.001
GH1mo vs. GH3mo	Z = 7.77, p < 0.001
GH3mo vs. GH6mo	Z = 7.77, p < 0.001
GH6mo vs. GH12mo	Z = 6.21, p < 0.001
GH12mo vs. GH2yrs	Z = 5.57, p < 0.001
Vpreop vs. V1mo	Z = 7.77, p < 0.001
V1mo vs. V3mo	Z = 7.77, p < 0.001
V3mo vs. V6mo	Z = 7.77, p < 0.001
V6mo vs. V12mo	Z = 7.77, p < 0.001
V12mo vs. V2yrs	Z = 5.16, p < 0.001
SFpreop vs. SF1mo	Z = 7.77, p < 0.001
SF1mo vs. SF3mo	Z = 7.77, p < 0.001
SF3mo vs. SF6mo	Z = 7.77, p < 0.001
SF6mo vs. SF12mo	Z = 7.77, p < 0.001
SF12mo vs. SF2yrs	Z = 5.51, p < 0.001
REpreop vs. RE1mo	Z = 7.77, p < 0.001
RE1mo vs. RE3mo	Z = 7.77, p < 0.001
RE3mo vs. RE6mo	Z = 7.77, p < 0.001
RE6mo vs. RE12mo	Z = 6.15, p < 0.001
RE12mo vs. RE2yrs	Z = 5.64, p < 0.001
MHpreop vs. MH1mo	Z = 7.77, p < 0.001
MH1mo vs. MH3mo	Z = 7.77, p < 0.001
MH3mo vs. MH6mo	Z = 7.77, p < 0.001
MH6mo vs. MH12mo	Z = 7.77, p < 0.001
MH12mo vs. MH2yrs	Z = 7.77, p < 0.001
VAS-LPpreop vs. VAS-LP1mo	Z = 5.51, p < 0.001
VAS-LP1mo vs. VAS-LP3mo	Z = 5.51, p < 0.001
VAS-LP3mo vs. VAS-LP6mo	Z =. 3.41, p < 0.001
VAS-LP6mo vs. VAS-LP12mo	Z = 1.34, p = 0.18
VAS-LP12mo vs. VAS-LP2yrs	-
VAS-BPpreop vs. VAS-BP1mo	Z = 7.77, p < 0.001
VAS-BP1mo vs. VAS-BP3mo	Z = 7.72, p < 0.001
VAS-BP3mo vs. VAS-BP6mo	Z = 5.3, p < 0.001
VAS-BP6mo vs. VAS-BP12mo	-
VAS-BP12mo vs. VAS-BP2yrs	-

In addition, the improvement in all indices was found to be irrespective of age (p > 0.14) (Table 5). When comparing the early versus late intervention groups, the difference in the various indices between the preoperative and two-year postoperative assessments was found to be statistically significant for RP (U_1,80 _= 574.5, p < 0.05), BP (U_1,80 _= 519, p < 0.01), GH (U_1,80 _= 504, p < 0.01), and MH (U_1,80 _= 493, p < 0.01). Interestingly, when examining collectively the age, gender, and time lapse from fracture to treatment, as predictors of outcome measures, multiple regression analysis demonstrated a main effect of time for RP (b = -0.27), GH (b = -0.39), and MH (b = -0.23) at a level of p < 0.05.

**Table 5:** Spearman rank order correlation coefficients rs. No values were found to be statistically significant at the level of p < 0.05. DIF: difference

	PF_DIF	RP_DIF	BP_DIF	GH_DIF	V_DIF	SF_DIF	RE_DIF	MH_DIF	VAS-LP_DIF	VAS-BP_DIF
Age	-0.102213	0.088234	0.101039	-0.095305	-0.153429	0.158809	0.026519	0.161395	-0.176885	-0.062734

## Discussion

Osteoporosis is a skeletal disease that is characterized by low bone mass and microarchitectural deterioration of bone tissue, leading to increased bone fragility and susceptibility to fracture [18]. The most common site for osteoporotic fractures is the spine (thoracolumbar junction), followed by the proximal femur and distal radius [3]. OVFs can result in significant morbidity [3]. Although most of these fractures may heal, 15-35% can lead to unacceptable outcomes, such as chronic back pain, spinal deformity, and neurologic deficit [3,19]. The emergence of these complications necessitates surgical management in order to rebuild stability of the spine and to improve QoL of affected individuals [8,11,20]. Treatment of OVFs is a factor that affects QoL and should be considered during management [21].

Delayed neurological deficits may occur from one week to 5.7 months after OVFs [22]. Predictive factors include burst fracture type, vacuum sign, kyphosis, angular instability, and retropulsion. In these cases, clinical symptomatology varies from low back pain and sciatica to cauda equina syndrome. The type of fracture (compression fractures), lack of surgical complications, and optimal restoration of retropulsion determined the significant improvement in clinical function after surgery for neurological deficits [22]. In our study, 44 patients underwent early surgical intervention (during the first month from the clinical emergence of fracture). The results of two parameters of SF-36 (RP and MH) were statistically significant between early and late surgical interventions.

Based on the literature, there are many studies evaluating PLF surgery with or without neural decompression for the management of OVFs. Akata et al. published a study of 14 patients being subjected to PLF without decompression due to OVFs, featuring albeit incomplete neurological deficits [23]. Their results demonstrated remarkable amelioration of back pain in conjunction with neurological improvement. Hence, the authors suggested that neural decompression may not be essential for the management of neurological deficits due to OVFs implementing dynamic mobilization and fixation of the fracture site [23]. Niu et al. also studied the efficacy of PLF in 24 patients with OVFs in the thoracolumbar junction with associated kyphosis [8]. Their outcomes were also favorable, demonstrating statistically significant improvement of perceived pain with regaining of the function recovery of enrolled individuals at a mean of 14.4 months within follow-up. Furthermore, the measure of the kyphotic Cobb angle was depicted to be remarkably decreased postoperatively [8]. In another retrospective investigation, Xu et al. studied 238 patients with old OVFs being subjected to PLF [1]. Recorded patient-reported outcome measures such as the VAS and Oswestry Disability Index (ODI) scores in conjunction with radiological parameters were significantly improved within follow-up, advocating for the favorable role of intervention in the overall QoL in affected individuals [1].

Kyphotic deformity in the osteoporotic spine can create an altered biomechanical environment, which may increase the incidence of compression vertebral fractures in adjacent unaffected segments [4]. The correction of kyphotic deformity and restoration of anatomic alignment represent core targets of PLF surgery. Moreover, the correction of deformity reduces the frequency of implant failure, which remains a significant problem in cases where instrumentation surgery is used to treat osteoporotic spine patients [24]. Alpantaki et al. stated that instrumentation should not end within the kyphotic segment in order to prevent screw loosening and junctional kyphosis [7]. Pull-out of fixed screws may occur when kyphosis is not restored [7]. Nakajima et al. noted that migration of pedicle screws occurred in three of 10 patients after PLF, stating also that bone mineral density (BMD) at the lumbar spine was significantly lower in patients with postoperative complications [5].

Our study presents the efficiency of PLF in patients with OVFs, emphasizing the impact on QoL. In this study, we decided to perform a more holistic evaluation of our patients, thus recruiting SF-36 for HRQoL analysis, evaluating not only perceived pain but also its associated impact on the psychosocial status of affected patients. All parameters of SF-36 presented significant improvement over the entire follow-up period, with VAS scores reaching a plateau at six months. The depicted improvement of these parameters proves the beneficial role of PLF in elderly patients who suffered from OVFs with or without referable neurological deficits. No major perioperative complications were recorded in the present study, advocating for the safety of the procedure even in this vulnerable population. However, the limitations of this study include the small sample size and the fact that this is not a randomized control trial study. In view of these limitations, future researchers could work toward these directions.

## Conclusions

Despite the fact that OVFs have a significant impact on the QoL of elderly patients, surgical treatment with PLF with or without decompression can lead to functional recovery, pain relief, and HRQoL amelioration. Our results demonstrated that the outcomes of PLF in the surgical treatment of these patients are remarkably favorable, demonstrating the safety and efficacy of the technique. However, further studies with larger sample sizes and more extended follow-up intervals are required in order to exclude safer conclusions about the precise role of this surgical technique in this vulnerable population.
